# Bone Marrow Metastasis Is an Early Stage of Bone Metastasis in Breast Cancer Detected Clinically by F18-FDG-PET/CT Imaging

**DOI:** 10.1155/2017/9852632

**Published:** 2017-08-13

**Authors:** Kusai M. Al-Muqbel

**Affiliations:** Department of Radiology and Nuclear Medicine, Faculty of Medicine, Jordan University of Science and Technology, Ramtha Street, Irbid 22110, Jordan

## Abstract

**Objective:**

To determine the value of 18F-FDG PET/CT in detection of bone marrow (BM) metastasis in breast cancer which is considered an early stage of bone metastasis.

**Patients and Methods:**

Retrospectively, breast cancer patients with bone metastasis were included. BM metastasis was considered if the lesion was PET positive/CT occult while bone metastasis was considered if the lesion was PET positive/ CT positive. BM metastases were observed sequentially on F18-FDG PET/CT.

**Results:**

We included 35 patients. Eighteen patients (51%) had BM metastases in addition to other bone metastases. BM metastases comprised 24% of all lesions. Posttreatment scan was performed on 26/35 patients. Twenty-three percent of BM metastases had resolved completely without causing bone destruction after treatment. Sixty-five percent of BM metastases had converted into bone metastases after treatment. Twelve percent of BM metastases had persisted after treatment.

**Conclusion:**

This retrospective study showed clinically by 18F-FDG PET/CT imaging that BM metastasis is an early stage of bone metastasis in breast cancer. Interestingly, 18F-FDG-PET/CT showed that early eradication of individual BM metastasis by systemic treatment precluded development of bone metastasis. However, more research is needed to study the impact of an early diagnosis of BM metastases on treatment outcome.

## 1. Introduction

A small number of breast cancer cells (BCCs) could exit the primary tumor site and enter the bone marrow (BM) during the early phase of tumor development. BCCs show a preference for the bone marrow. Immediately after BCCs migration and invasion into the BM, they interact with mesenchymal stem cells which protect BCCs from immunosurveillance. Moreover, these cells become dormant as they can remain in cycling quiescence close to the endosteum area in the BM. Quiescence of breast cancer cells (dormant cells) makes it increasingly difficult to target the dormant cancer cells by chemotherapy [1].

Anatomically, BCCs frequently metastasize to axial bone skeleton, that is, the spine, ribs, girdles, and bony pelvis. In adults, axial bone skeleton contains the red marrow, which provides vital factors for the BCCs creating what is called “bone metastatic niche.” These factors include ample cells, extracellular matrix, nutrition, and signaling molecules [2]. Bone marrow macrometastasis appears once the dormant BM metastatic cells outgrow (proliferate). Conventional bone marrow biopsies indicate that about 26–40% of patients with metastatic breast cancer have bone marrow involvements [3, 4].

In osteolytic bone metastasis, a complicated molecular interaction (called “vicious cycle” of molecular crosstalk) takes place between metastatic BCCs and bone metastatic niche. During this interaction, a variety of cytokines and growth factors are produced by metastatic BCCs which directly stimulate the osteoclast maturation or indirectly promote osteoclast differentiation. The latter is usually accomplished by stimulating the BM osteoblasts to produce Interlukin-6 (IL-6) and receptor activator of nuclear factor-kB ligand (RANKL) [1]. The survival and proliferation of metastatic BCCs in osteolytic bone metastasis are in turn promoted by several factors released by bone matrix resorption caused by osteoclast activation. These factors include transforming growth factor-*β* (TGF-*β*) and insulin-like growth factor-1 (IGF1) [2].

F18-FDG-PET/CT is a sensitive molecular imaging modality capable of diagnosing bone marrow metastases by means of increased FDG uptake in growing metastatic cancer cells [5]. In addition, F18-FDG-PET/CT is sensitive in detecting metastatic bone lesions, particularly osteolytic and mixed lesions [6]. However, until now, few studies have evaluated the BM metastases by F18-FDG-PET/CT in breast cancer. Our study aims to determine the value of F18-FDG-PET/CT in the diagnosis of bone marrow (BM) metastasis in breast cancer patients which is considered an early stage of bone metastasis.

## 2. Patients and Methods

The medical records of breast cancer patients with metastatic disease were reviewed retrospectively from January 2012 to June 2015. The study was approved by hospital IRB. We included in our study the patients who had metastatic bone disease proven by either staging or follow-up F18-FDG-PET/CT.

Bone marrow metastasis was considered if there was PET positive/CT occult lesion, that is, focal F18-FDG uptake on PET images overlying intact bone on CT images. This is in contrast to bone metastasis which is focal F18-FDG uptake on PET images overlying destructive bone lesion (osteolytic, osteoblastic, or mixed) on CT images [5, 6]. Bone marrow lesions were observed sequentially in the patients who had undergone sequential F18-FDG-PET/CT, that is, pretreatment and posttreatment F18-FDG-PET/CT scans. Posttreatment assessment was categorized as responsive, progressive, or stable based on FDG focal uptake. Disappearance of FDG focal uptake posttreatment was considered responsive, increasing FDG focal uptake (in terms of intensity and/or number), posttreatment was considered progressive and stable disease was considered if no change was noted in posttreatment FDG focal uptake.

Included patients in this study were referred for F18-FDG-PET/CT for staging of breast cancer, for follow-up of breast cancer, and/or for posttreatment evaluation of metastatic disease. The patients received systemic chemotherapy and/or hormonal therapy according to current international guidelines.

### 2.1. Imaging

F18-FDG-PET/CT imaging was acquired utilizing an integrated PET/CT device (Discovery 600; GE Medical Systems, Milwaukee, Wis). The whole-body mode (from the base of the skull down to upper thighs) was implemented as the standard software. Before the PET/CT acquisition, the patients fasted for at least 6 hours. All patients were tested to confirm that their glucose level was not more than 200 mg/dL before F18-FDG administration. Before PET, unenhanced CT was performed according to a standardized protocol performed with the following settings: transverse 2.5 mm section thickness, 120 kVp, and 80–180 mA according to local body thickness. PET scans were obtained 40–90 minutes after an intravenous administration of mean 296 Mbq (8 mCi) F18-FDG. The acquisition time was 2-3 minutes per bed position in the two-dimensional mode. Images were reconstructed with attenuation-weighted ordered-subset expectation maximization with and without attenuation correction.

## 3. Results

**Figure 1:**
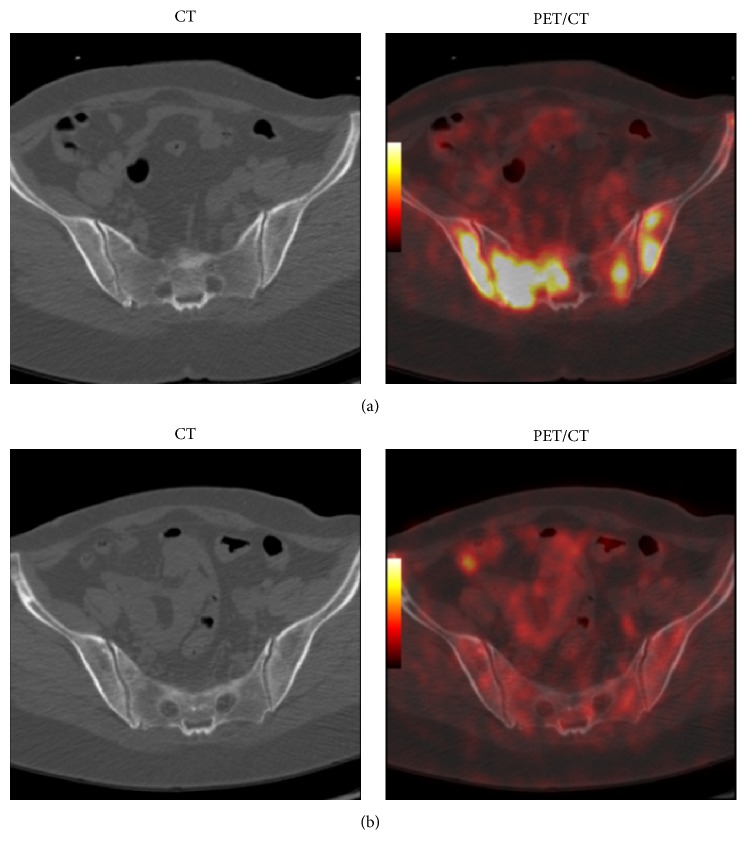
Breast cancer patient (40 years old) evaluated by F18-FDG-PET/CT showing bone marrow metastases on baseline scan (a) which was eradicated by chemotherapy before causing bone destruction as shown on 3 mo posttreatment scan (b).

Thirty-five patients, with an average age of 48.1 y (ranging between 27 and 80 years), were included in our study. Twenty patients were newly diagnosed with breast cancer metastasized to bone while 15 patients had developed bone metastasis several years after diagnosis of breast cancer (Table 1). Eighteen patients (51%) had BM metastases (ranging between 2 and 70 lesions with average of 23 lesions) (Figure 1) in addition to other structural (destructive) bone metastatic lesions (ranging between 1 and 110 lesions with average of 33) (Table 1). BM metastases comprised 24% of all metastatic lesions noted on pretreatment F18-FDG-PET/CT according to the following formula: BM lesions/(BM lesions + bone lesions) (Table 2).

Twenty-six out of 35 patients had undergone 3–10 months' posttreatment F18-FDG-PET/CT. Two out of 35 patients had been lost to follow-up at our hospital. Seven out of 35 patients were severely ill secondary to development of disseminated metastatic disease (BM, bone, liver, lung, and lymph nodes) several years after breast cancer diagnosis. Accordingly, they died within 2-3 months afterward, so they had no follow-up. Of note, they had had large number of BM lesions (Table 1).

In 26 patients who had follow-up, only 4% of metastatic lesions were BM lesions and 96% of the lesions were bone metastases (Table 2). Eighteen out of 26 patients (69%) had complete response after treatment as all BM lesions had disappeared and all bone metastatic lesions had become PET negative. Five out of 26 patients (19%) had no response to treatment with disease progression as PET positive lesions had increased in number including BM lesions and/or bone metastatic lesions. Three out of 26 patients (12%) had stable disease or had partial response to treatment as PET positive lesions had been stable or had decreased in number, respectively (Table 1).

BM lesions had totally disappeared in responsive patients. In 5 progressive patients, 2 patients had new BM lesions, 1 patient had her BM lesions increased in number, 1 patient had her BM lesions decreased in number as they had progressed into bone metastases, and 1 patient had partial response as her BM lesions had partially disappeared (Table 1).

**Figure 2:**
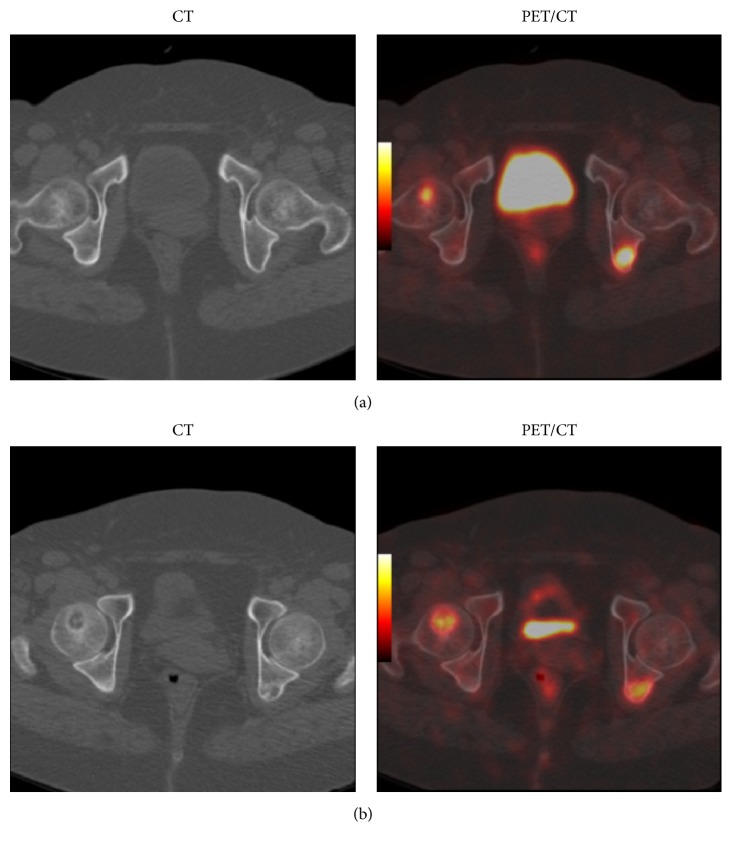
Breast cancer patient (51 years old) evaluated by F18-FDG-PET/CT showing two bone marrow metastatic lesions on baseline scan (a) which progressed to FDG-positive osteolytic metastatic bone lesions on 3 mo follow-up scan (b).

**Figure 3:**
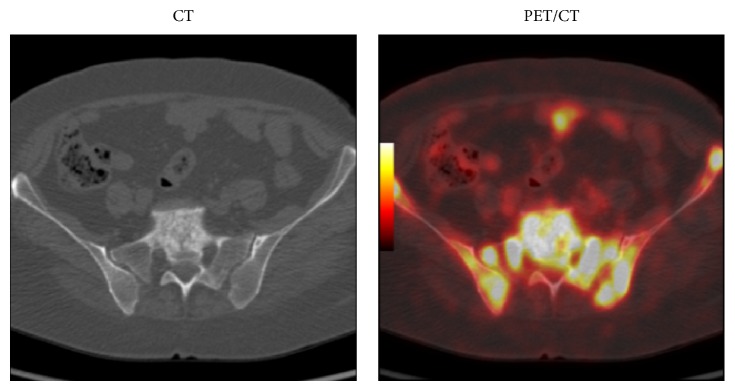
Breast cancer patient (35 years old) evaluated by pretreatment F18-FDG-PET/CT showing multiple bone marrow metastatic lesions in pelvic bones with concomitant early development of osteoblastic metastatic bone lesion in S1 vertebral body.

Twenty-three percent of BM metastases (17 out of 75 lesions) had resolved completely without causing bone destruction after treatment as noted on posttreatment 18F-FDG-PET/CT (Figure 1). Sixty-five percent of BM metastases (49 out of 75 lesions) had converted into structural destructive bone metastatic lesions in those patients who underwent posttreatment 18F-FDG-PET/CT. The structural destructive bone metastatic lesions were mostly osteolytic/mixed lesions (Figure 2) and less frequently osteoblastic (Figure 3). Twelve percent of BM metastases (9 out of 75 lesions) had persisted on posttreatment F18-FDG-PET/CT (Table 1).

## 4. Discussion

Bone marrow and bone receive a high volume of blood flow and both are rich in growth factors. It is well known that BCCs spread hematogenously. Ninety percent of bone metastases in breast cancer patients start as intramedullary BCCs deposits in the red marrow [3, 4]. The deposited BCCs in the red marrow usually go in quiescence escaping immunosurveillance and chemotherapy. Months or years later, the quiescent BCCs overgrow and become BM macrometastasis. The latter will interact with BM microenvironment. Such interaction eventually results in the formation of bone metastasis and it is regulated by various growth factors among the tumor, osteoclasts, and osteoblasts. Interestingly, bone metastasis was observed in more than 70% of patients with advanced breast cancer as bone microenvironment is suitable for the growth of metastatic BCCs [7]. Bone marrow carcinosis is not always associated with radiographic abnormality [3]. F18-FDG-PET/CT is highly sensitive in detecting bone marrow metastasis and osteolytic bone metastases [5, 6]. On the other hand, CT is not able to detect early BM metastasis (CT occult lesions) even when utilizing the optimal CT window width and level [4].

Bone marrow metastases were noted clinically on F18-FDG-PET/CT in about half of our patients who presented with new bone metastases. Our data showed that bone marrow metastases played a significant role in metastatic bone disease pathogenesis as noted clinically in pre- and posttreatment F18-FDG-PET/CT. This study showed that molecular imaging (FDG-PET scanning) but not CT scanning has a capability to detect BM metastasis. This study showed by clinical molecular imaging that osteolytic and osteoblastic metastatic bone lesions were preceded by bone marrow metastases. In other words, we showed clinically that BM metastasis is an early stage of bone metastasis that is detected by F18-FDG-PET but not by CT. Interestingly, we showed that early successful eradication of bone marrow metastatic lesion by the systemic treatment precluded the development of destructive metastatic bone lesion as 24% of the observed BM lesions in our patients had disappeared without causing bone destructive lesions on follow-up.

However, it is not necessary to find concomitant BM metastasis with newly diagnosed bone metastasis in every breast cancer patient by F18-FDG-PET/CT as half of our patients had no BM metastasis. This is justifiable because BM metastasis detection by molecular imaging is time dependant. The probability of detecting BM metastasis by molecular imaging is high if the patient is imaged at the beginning of bone metastatic process. More time elapsed after the beginning of bone metastatic process means that few or no BM metastatic lesions are detected by molecular imaging.

In one study utilizing F18-FDG-PET/CT, 17 breast cancer patients were found on restaging F18-FDG-PET/CT to have bone marrow metastases concomitant with bone metastases causing 57% stage upgrade. The early identification of BM metastases in this study had a direct consequence on the choice of the therapeutic approach. They showed that one patient with bone marrow metastases had better prognosis due to the early beginning of the systemic therapy [5].

Another case report showed biopsy proven diffuse bone marrow carcinosis in recurrent breast cancer patient by F18-FDG-PET/CT. Interestingly, this patient had the phenomenon of SuperScan on bone scintigraphy, which is thought to be secondary to high bone turnover stimulated by diffuse bone marrow carcinosis which was reversible after aggressive treatment [4].

To the best of our knowledge, this is the first clinical report demonstrating the role of bone marrow in the pathogenesis of bone metastases in breast cancer by tracking bone marrow metastases clinically on sequential F18-FDG-PET/CT on several patients. This study confirms clinically by molecular imaging the relation between BM metastases and bone metastases. This in turn leads to the fact that F18-FDG-PET/CT could be helpful in early diagnosis of bone metastasis particularly in high-risk breast cancer patients (i.e., young patients, locally advanced disease, and inflammatory breast cancer). Accordingly, treatment can be started early leading to potentially better outcome.

It is important to differentiate between diffuse bone marrow carcinosis and focal bone marrow metastases. Anemia was the most frequent symptom at presentation in patients with diffuse bone marrow carcinosis which is associated with poor prognosis according to one study [8]. Median survival time after the diagnosis of apparent diffuse bone marrow carcinosis was found to be 6.43 months in that study [8]. In contrast, the estimated median overall survival from the time of diagnosis of diffuse bone marrow carcinosis was 19 months in another study, thereby emphasizing the fact that diagnosis of diffuse bone marrow carcinosis should not be regarded as a poor prognostic indicator with possible achievement of long-lasting disease control by systemic treatment [9]. In our study, 7 patients had disseminated BM metastasis as part of disseminated visceral, nodal, and BM/bone metastasis. Those patients had bad prognosis as they died within 2-3 months after presentation. Those patients presented with disseminated disease relapse several years after breast cancer diagnosis (range of 1–6 years, average 3.3 years).

This study is limited by being retrospective. This is true because bone marrow metastasis is a temporary state of bone metastasis. In other words, detection of BM metastasis is affected by when to do the molecular scan. We admit that most patients who were found to have bone marrow metastases by molecular imaging already had bone metastases somewhere else in their bony skeleton. Within the limitation of this retrospective study, we do not know for sure whether early detection of bone marrow metastases by molecular imaging will have a significant impact on patients' management/prognosis or not.

## 5. Conclusion

This retrospective study showed clinically by F18-FDG-PET/CT imaging that bone marrow metastasis is an early stage of bone metastasis in breast cancer preceding osteolytic and osteoblastic metastatic bone lesions. Interestingly, F18-FDG-PET/CT showed that early eradication of individual bone marrow metastasis by systemic treatment precluded development of destructive bone metastasis. However, more research is needed to study the impact of an early diagnosis of bone marrow metastases by molecular imaging on breast cancer treatment outcome.

## Figures and Tables

**Table 1:** Structural and functional features of metastatic bone disease in 35 breast cancer patients as noted on F18-FDG-PET/CT scans both at baseline (pretreatment) for all patients and at follow-up (posttreatment) for 26 patients.

No	Age (y)	Interval (y)^*∗*^	Baseline F18-FDG PET/CT scan		Interval (mo)^*∗∗*^	Follow-up F18-FDG PET/CT scan		
			BM lesions (PET)^*ǂ*^	Bone lesions (PET/CT)^*¥*^		BM lesions (PET)^*ǂ*^	Bone lesions (PET/CT)^*¥*^	Posttreatment response
1	80	0	14	44/44	—	—	—	—
2	38	0	6	16/16	3	0	0/19	Responsive
3	42	0	16	90/90	—	—	—	—
4	54	0	8	28/28	6	0	0/34	Responsive
5	42	0	24	110/110	4	0	0/130	Responsive
6	61	0	0	40/48	7	0	0/48	Responsive
7	42	0	0	35/43	6	0	0/43	Responsive
8	40	0	0	15/15	6	0	0/15	Responsive
9	54	0	0	40/40	4	0	40/40	Stable
10	40	0	0	9/9	7	0	0/9	Responsive
11	38	0	0	23/27	4	9	40/45	*Progressive*
12	55	0	0	12/12	10	0	0/12	Responsive
13	50	0	15	40/40	5	0	0/50	Responsive
14	55	0	0	88/88	5	0	0/88	Responsive
15	60	0	0	101/101	3	0	0/101	Responsive
16	60	0	0	79/79	6	0	79/79	Stable
17	45	0	0	65/65	6	0	0/65	Responsive
18	42	0	8	40/40	3	5	45/45	Stable
19	56	0	0	0/17	10	0	10/63	*Progressive*
20	32	0	0	3/3	6	0	0/3	Responsive
21	60	5	22	76/76	—	—	—	—
22	61	2	70	0/0	—	—	—	—
23	56	6	65	67/67	—	—	—	—
24	55	4	28	35/35	—	—	—	—
25	27	3	45	26/26	—	—	—	—
26	43	2	68	45/52	—	—	—	—
27	47	1	17	10/12	—	—	—	—
28	45	2	3	6/6	6	0	0/7	Responsive
29	40	3	0	0/1	6	0	0/1	Responsive
30	39	0.5	2	25/25	6	19	47/47	*Progressive*
31	45	7	3	4/6	7	0	0/8	Responsive
32	55	6	6	23/23	6	2	56/56	*Progressive*
33	54	0.5	0	0/13	9	14	60/81	*Progressive*
34	48	2	0	8/8	3	0	0/8	Responsive
35	48	3	0	88/88	3	0	0/88	Responsive

*Total*			*420*	*1353*		*49*	*1185*	

BM = bone marrow metastatic lesions, *∗* = interval between tissue diagnosis of breast cancer and diagnosis of bone/bone marrow metastasis by F18-FDG-PET/CT scan, *∗∗* = interval between baseline and follow-up F18-FDG-PET/CT scans, *ǂ* = PET positive/CT occult lesions (BM lesions), and ¥ = PET positive/CT positive lesions (i.e., number of bone lesions seen on CT that are hypermetabolic on PET). Example: patient # 2 had 6 BM lesions (PET +ve/CT −ve) and 16 active bone lesions (PET +ve/CT +ve) on baseline. On follow-up, she had 19 inactive bone lesions (PET −ve/CT +ve) and all BM lesions had disappeared (3 lesions had become bone metastasis and 3 had resolved without causing bone destruction). F18-FDG PET/CT = fluorine-18 fluoro-2-deoxy-D-glucose positron emission tomography/computed tomography.

**Table 2:** Number and percentage of BM metastatic lesions and bone metastases (osteolytic/mixed/osteoblastic) in 35 patients noted on pretreatment F18-FDG-PET/CT scans and in 26 patients noted on posttreatment F18-FDG-PET/CT.

	BM (PET)	Bone metastases (CT)	Total (PET/CT)	Patients (number)
PET/CT1 (number of lesions)	420	1353	1773	35
PET/CT1 (% of lesions)	24	76	100	35
PET/CT2 (number of lesions)	49	1185	1234	26
PET/CT2 (% of lesions)	4	96	100	26

BM = bone marrow, PET/CT1 = pretreatment F18-FDG-PET/CT, PET/CT2 = posttreatment F18-FDG-PET/CT, and F18-FDG PET/CT = fluorine-18 fluoro-2-deoxy-D-glucose positron emission tomography/computed tomography.
